# A new species of *Strongylacron* (Copepoda, Harpacticoida, Cletodidae) from intertidal mudflats in the Korean peninsula

**DOI:** 10.3897/zookeys.617.7600

**Published:** 2016-09-15

**Authors:** Jong Guk Kim, Tae Won Jung, Seong Myeong Yoon

**Affiliations:** 1 Department of Marine Life Science, College of Natural Sciences, Chosun University, Gwangju 61452, Korea; 2 National Marine Biodiversity Institute of Korea, Seocheon 33662, Korea; 3 Department of Biology, College of Natural Sciences, Chosun University, Gwangju 61452, Korea

**Keywords:** Cletodidae, glabrum, harpacticoid, Korea, new species, Strongylacron, taxonomy

## Abstract

A new species, *Strongylacron glabrum* **sp. n.** is described from intertidal mudflats in the Korean peninsula. The new species is assigned to the monospecific genus *Strongylacron* Gee & Huys, 1996 in accordance with the generic morphological features of the rostrum, antennary exopod, and thoracic legs. However, *Strongylacron glabrum* **sp. n.** is clearly distinguished from the type species, *Strongylacron buchholtzi* (Boeck, 1873), by the naked anterior margin of rostrum, the elongate exopod and endopodal lobe of female P5 approximately 3.5 and 2.7 times as long as width, respectively, and the presence of 8–10 rod-like projections on prosomites. The genus *Strongylacron* is first recorded from Korean waters by the present study.

## Introduction

The harpacticoid copepod genus *Strongylacron* Gee & Huys, 1996 belonging to the family Cletodidae T. Scott, 1904 was established by Gee and Huys (1996) as a part of an effort to resolve the relationship among members of *Enhydrosoma* Boeck, 1873. The latter genus was recognized as a heterogeneous group by Fiers (1987) and Mielke (1990). After the studies of Lang (1936, 1948), this genus was distinguished from *Cletodes* Brady, 1972 by subtle morphological characteristics such as the form and setation of the distal segment on legs 3 and 4 (Gee 1994, 2001; Gee and Huys 1996). In the revision of *Enhydrosoma*, Gee (1994) first proposed the *buchholtzi*-species group representing differences from others in the rostrum, antenna, maxillule and male P3 endopod. In addition, he suggested that this group should be removed from *Enhydrosoma*. Thereafter, the *buchholtzi*-species group was revised and the following three genera were established: *Schizacron* Gee & Huys, 1996, *Spinapecruris* Gee, 2001, and *Strongylacron* Gee & Huys, 1996 (Gee 1994, 2001; Gee and Huys 1996). *Strongylacron* is a monospecific genus containing *Strongylacron buchholtzi* (Boeck, 1873) (Walter and Boxshall 2016). The type species has been chiefly reported from European waters and occurs usually around estuary sediments containing high organic content (Boeck 1873; Sars 1909; Wells 1963; Gee and Huys 1996).

In South Korea, ten cletodid harpacticoids have been reported from various environments: Lee and Chang (2007) reported three species, *Limnocletodes behningi* Borutzky, 1926, *Limnocletodes angustodes* Shen & Tai, 1963, and *Kollerua longum* (Shen & Tai, 1979), from salt marshes and estuaries; Kim (2013) recorded *Enhydrosoma curticauda* Boeck, 1873 from coral sands in Udo, Jeju Island; six species, *Enhydrosoma coreana* Kim, Trebukhova, Lee & Karanovic, 2014, *Enhydrosoma apimelon* Karanovic, Kim & Lee, 2015, *Enhydrosoma robustum* Karanovic, Kim & Lee, 2015, *Enhydrosoma kosmetron* Karanovic, Kim & Lee, 2015, *Geehydrosoma intermedia* (Chislenko, 1978), and *Paracrenhydrosoma kiai* Song, Dahms, Lee, Ryu & Khim, 2014, were recently reported from intertidal and subtidal muddy bottoms (Kim et al. 2014; Song et al. 2014; Karanovic et al. 2015).

While studying harpacticoid copepods from Korean waters as a part of the ‘Survey of indigenous biological resources of Korea’, a new harpacticoid copepod belonging to the genus *Strongylacron* was discovered and reported here as *Strongylacron glabrum* sp. n. along with detailed description and illustrations.

## Materials and methods

Sampling was performed with a sieve of 212 µm mesh from intertidal mudflats on the south-western coasts of Korea. In ebb tides, surface sediments (< 5 cm sediments depth) were obtained by using large spoons. Samples remaining on sieve were fixed initially with 5% formaldehyde-seawater solution. Harpacticoid specimens were preserved with 99.9% ethanol after sorting in the laboratory. They were dissected by using tungsten needles under stereo microscope (Discovery, V8; Carl Zeiss, Germany) and then mounted on polyvinyl lactophenol or lactophenol. The observations and drawings were performed by light microscope (ECLIPSE 80*i*; Nikon, Japan) equipped with a drawing tube. Several specimens were examined in a scanning electron microscope (SEM). They were cleaned by an ultrasonic machine, prefixed by 4% glutaraldehyde, postfixed by 2% OsO_4_, dehydrated through graded ethanol solutions, air-dried, and coated with gold. The dried materials were observed under SEM (VEGA 3 LM; Tescan, Czech Republic), with an accelerating voltage of 20 kV and working distances between 17.90–20.50 mm. Descriptions and line drawings examined under 400–1,000× magnifications were made based on the paratypes. All materials examined were deposited in Chosun University and the National Institute of Biological Resources
(NIBR), Korea.

The terminology of the body and appendage morphology follows Huys and Boxshall (1991). Abbreviations used in the text and figures are:

ae
 aesthetasc 


exp
 exopod 


enp
 endopod 


exp (enp)-1 (2, 3)
 to denote the proximal (middle, distal) segment of a three-segmented ramus 


P1-P6
 first to sixth thoracic legs 


## Systematic accounts

### Family Cletodidae T. Scott, 1904 Genus *Strongylacron* Gee & Huys, 1996

#### Strongylacron glabrum sp. n.

Taxon classificationAnimaliaHarpacticoidaCletodidae

http://zoobank.org/A16A2690-3712-4FDC-A7CD-067CD78D8B25

Figs 2 , 3 , 4 , 5 , 6 , 7 , 8 , 9 , 10

##### Type locality.

**Figure 1.**
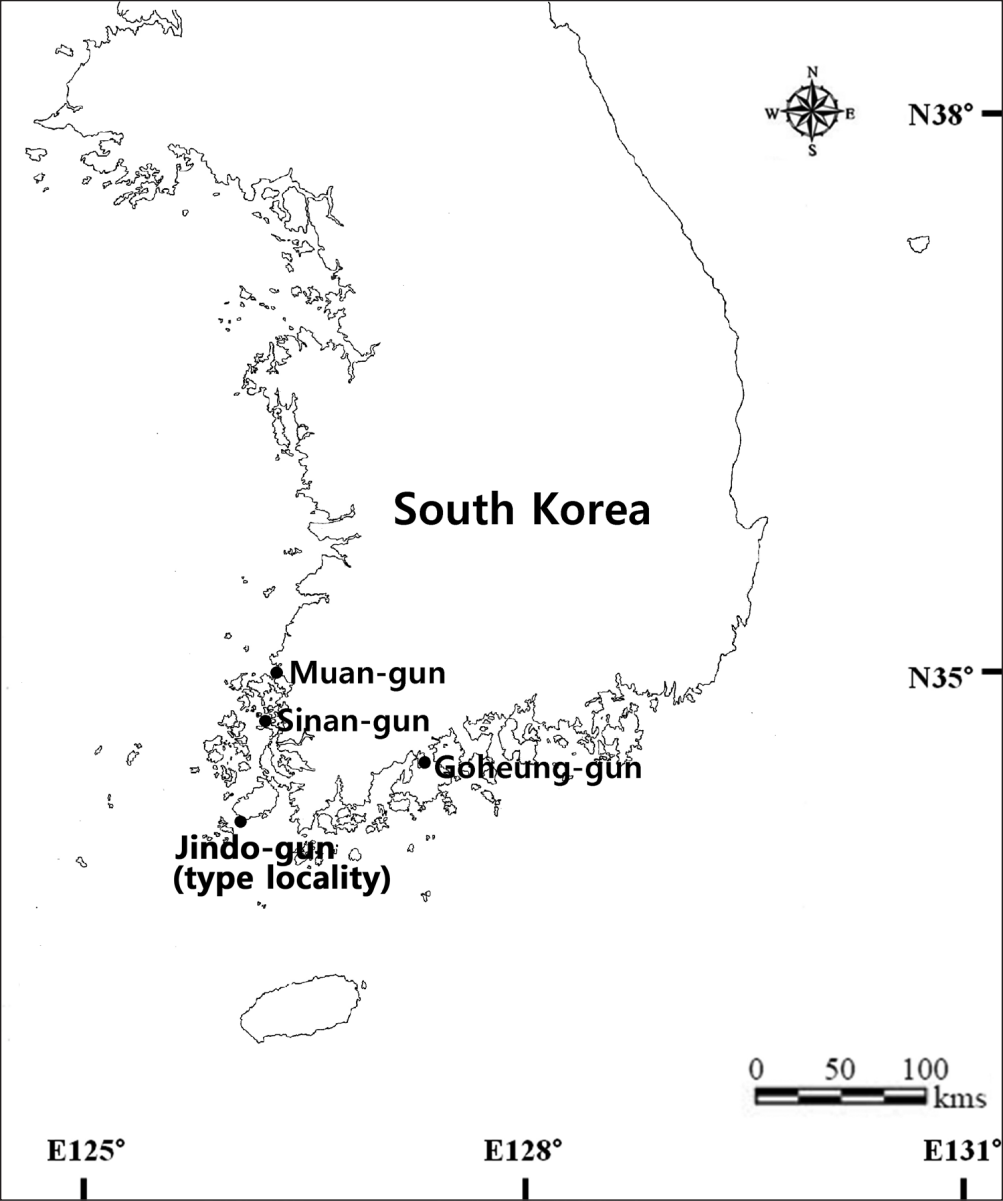
Localities of the sampling stations of the present study in South Korea.

South Korea, Jeollanam-do Province: Jindo-gun County, Imhoe-myeon, Namdong-ri, 34°21.666'N, 126°09.449'E, intertidal mudflats (Fig. 1).

##### Material examined.

Holotype ♀ (NIBRIV0000326503) and allotype ♂ (NIBRIV0000326504), both undissected and preserved in 99.9% ethanol. Paratypes: 2♀♀ (NIBRIV0000326505, NIBRIV0000326506) and 2 ♂♂ (NIBRIV0000326507, NIBRIV0000326508) dissected and mounted on each slide, respectively. All materials were collected from mud flats on type locality on 9 April 2013.

##### Additional material.

South Korea, Jeollanam-do Province: 15 ♀♀, Muan-gun County, Haeje-myeon, Songseok-ri, 35°9.477'N, 126°20.864'E, 9 April 2013; 2 ♀♀, Goheung-gun County, Gwayeok-myeon, Noil-ri, 34°41.275'N, 127°19.447'E, 30 April 2013. For the photographs of SEM: 4 ♀♀ and 2 ♂♂, Jeollanam-do Province, Sinan-gun County, Aphae-myeon, Sinyong-ri, 34°52.990'N, 126°17.867'E, 6 April 2012. All materials collected from sediments composed of mud or muddy sand on each locality (Fig. 1).

##### Diagnosis.

Habitus semi-cylindrical, approximately 900 µm; each prosomite with 8 or 10 rod-like projections. Rostrum fused to cephalothorax basally, slightly recurved dorsally; anterior margin rounded and naked. Genital field with vestigial P6 represented by seta. Caudal rami approximately 2.5 times (female) and 3.6 times (male) as long as width; tube pore on outer margin inserted proximally; caudal seta VI shorter than seta IV. Antennary endopod with stout spine-like seta at distal corner. Mandibular gnathobase with 3 bicuspid teeth, without seta; basis with 2 short and 1 long setae. Female P5 exopod 3.5 times as long as width; innermost seta on exopod shorter than exopod in length; endopodal lobe approximately 2.7 times as long as width. Male P5 endopodal lobe, outer most seta on endopodal lobe reaching half of middle seta on exopod.

##### Female.

**Figure 2.**
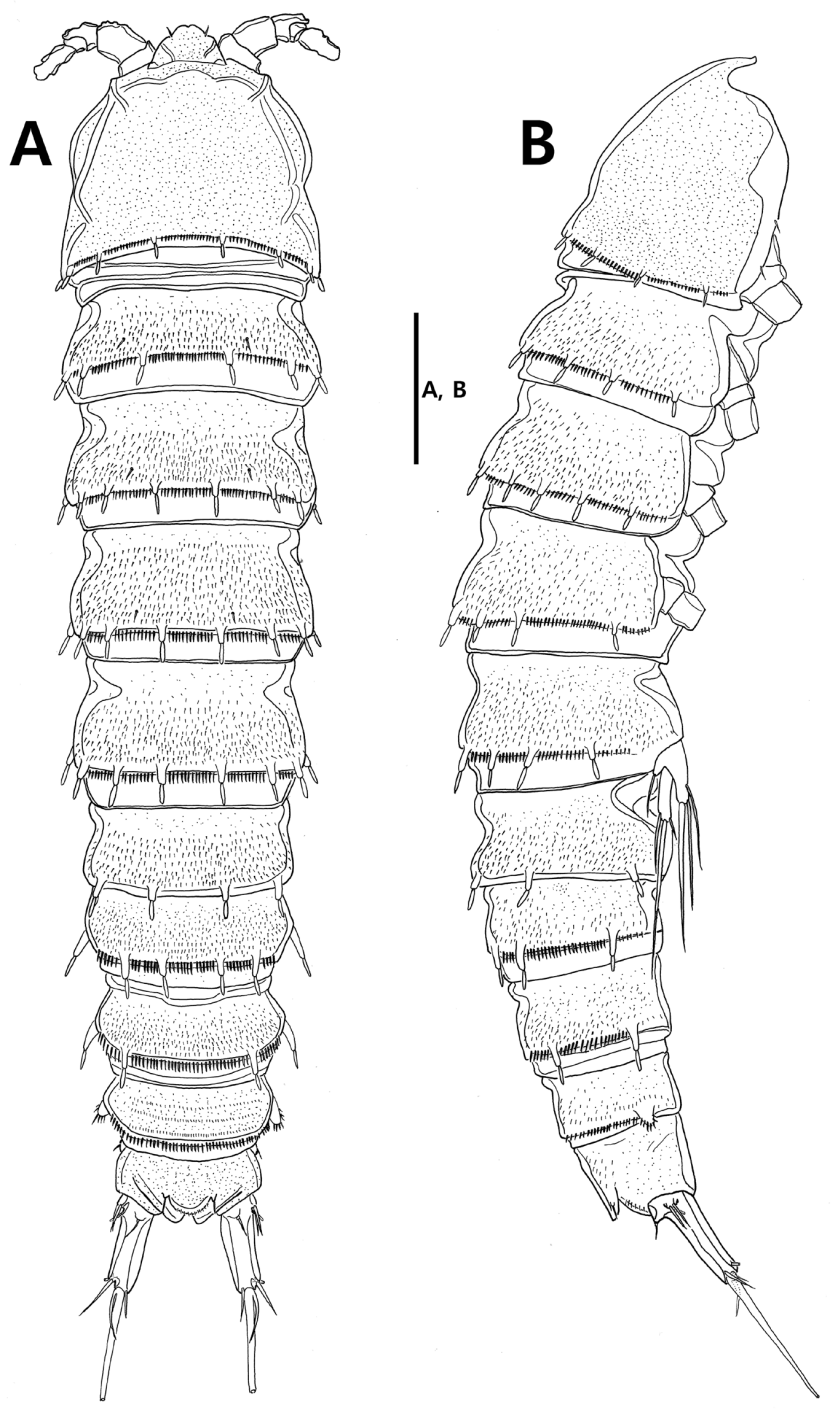
*Strongylacron glabrum* sp. n. female. **A** habitus, dorsal **B** habitus, lateral. Scale bar 100 µm.

**Figure 3.**
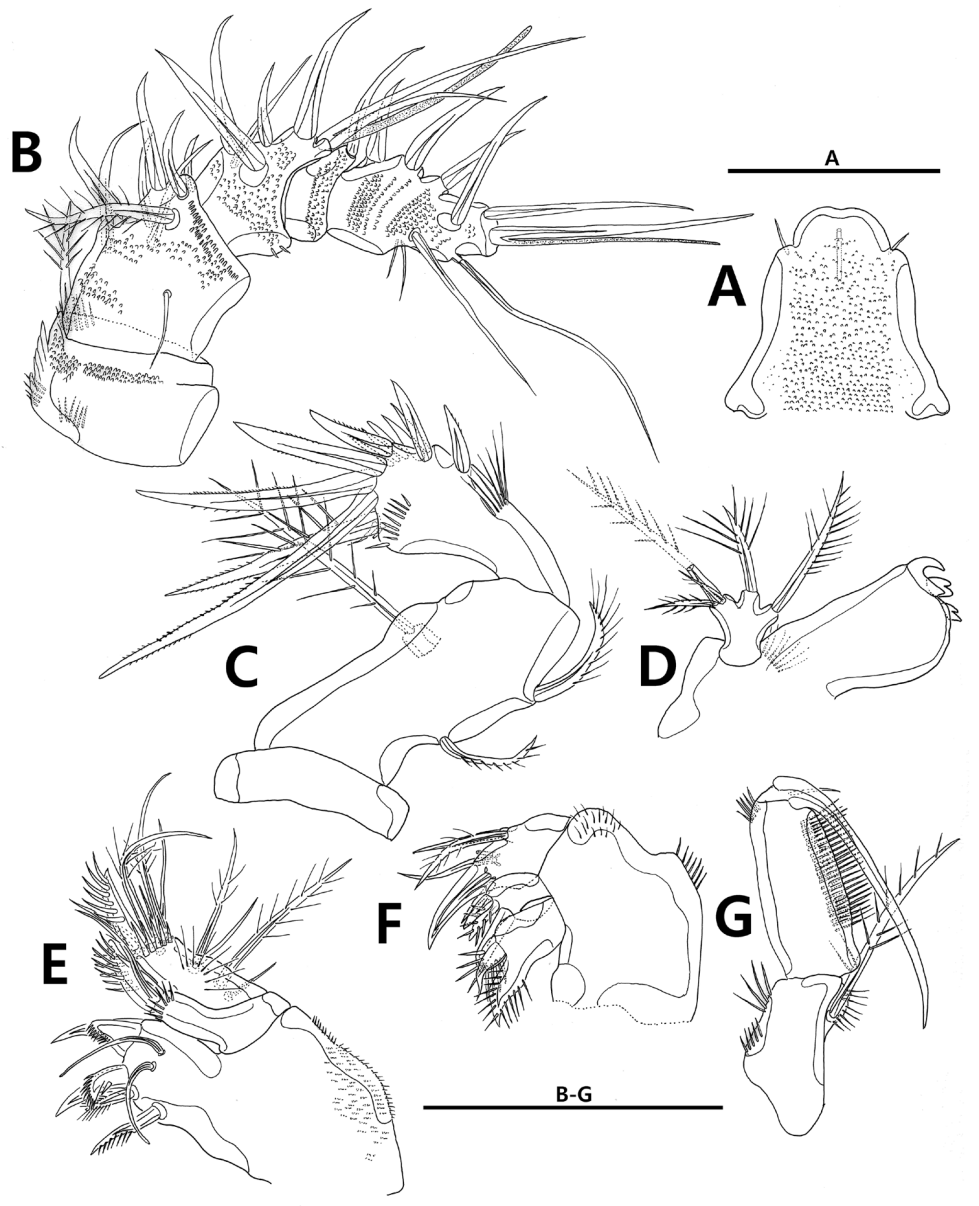
*Strongylacron glabrum* sp. n. female. **A** rostrum **B** antennule **C** antenna **D** mandible **E** maxillule **F** maxilla **G** maxilliped. Scale bars 50 µm.

Body (Figs 2A, B, 8A) semi-cylindrical, tapering posteriorly, with inconspicuous boundary between prosome and urosome; total length including tip of rostrum and caudal rami from 889.1 to 923.1 µm (mean 905.3 µm, *n* = 3) in lateral view. All somites covered with fine setules on surface; posterior border with row of setules except for anal somite. Rostrum (Figs 3A, 8E) well-developed, fused to cephalothorax basally, slightly recurved dorsally, with pair of subapical sensilla; anterior margin rounded, naked, slightly concave midway; posterior surface with tube pore.

Prosome (Figs 2A, B, 8B–D) 4-segmented, comprising cephalothorax and 3 free pedigerous somites. Cephalothorax slightly shorter than succeeding somites combined, with 8 rod-like projections. Each posterior border of 3 free somites (Fig. 8C) with 8, 10, 8 rod-like projections bearing sensillum, respectively.

**Figure 4.**
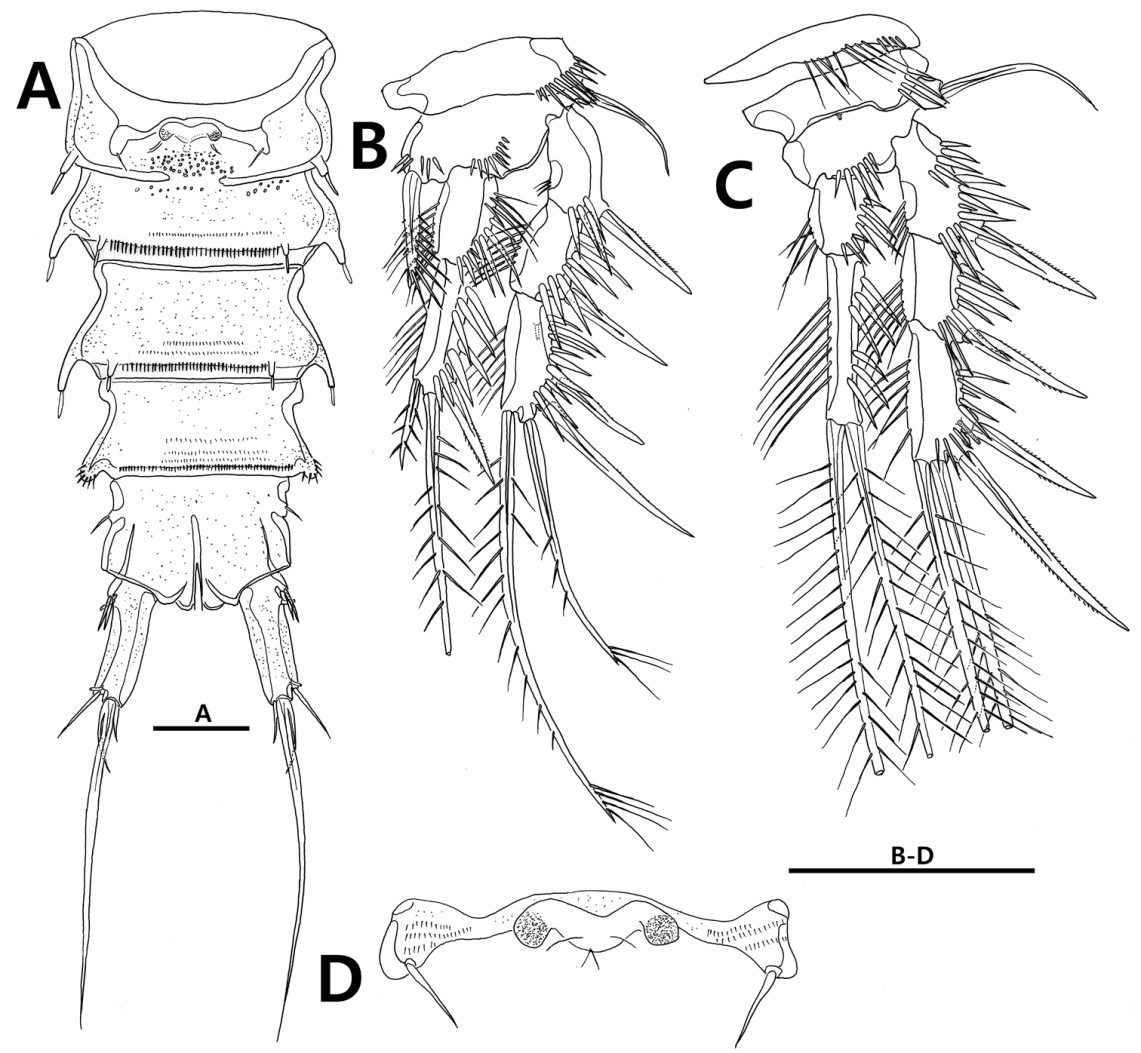
*Strongylacron glabrum* sp. n. female. **A** urosome except P5-bearing somite, ventral **B** genital field **C** P1 **D** P2. Scale bars 50 µm.

Urosome (Figs 2A, B, 4A) 5-segmented, comprising P5-bearing somite, genital double-somite, and 3 postgenital somites. P5-bearing somite with 8 rod-like projections bearing sensillum on posterior border dorsally. Genital double-somite, dorsal and lateral surfaces completely divided by suture, but ventral surface (Fig. 4A) partially fused; each posterior border with 6 and 8 rod-like projections bearing sensillum, respectively. Genital field (Fig. 4D) with vestigial P6 represented by seta. Urosomite 4 with 6 rod-like projections bearing sensillum on posterior margin. Urosomite 5 with pair of lateral protrusions covered with setules. Anal somite (Fig. 6D) with semi-circular operculum bearing pair of setae and 1 row of setules on posterior margin; lateral margin of each side with extra tube pore.

**Figure 5.**
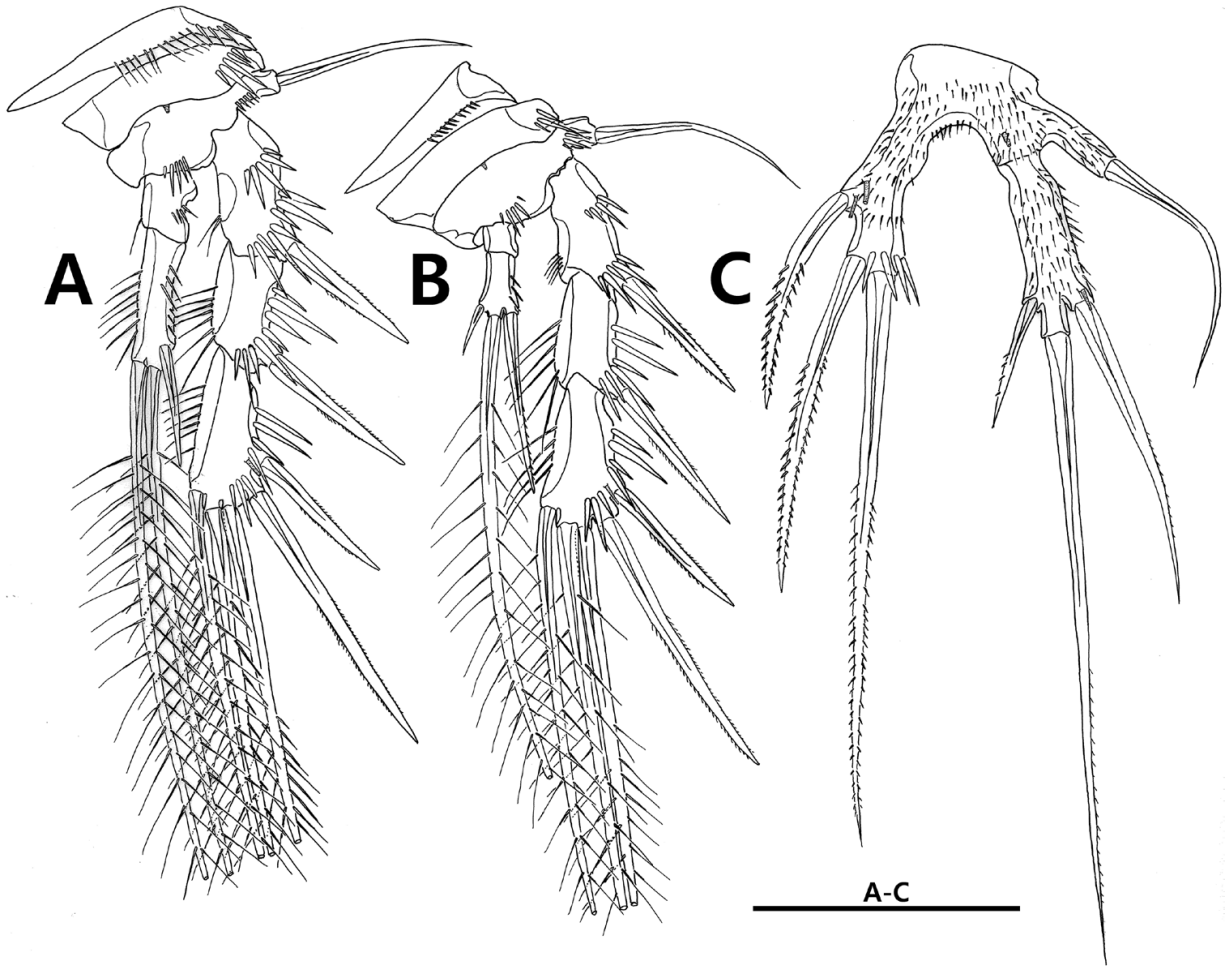
*Strongylacron glabrum* sp. n. female. **A** P3 **B** P4 **C** P5. Scale bar 50 µm.

**Figure 6.**
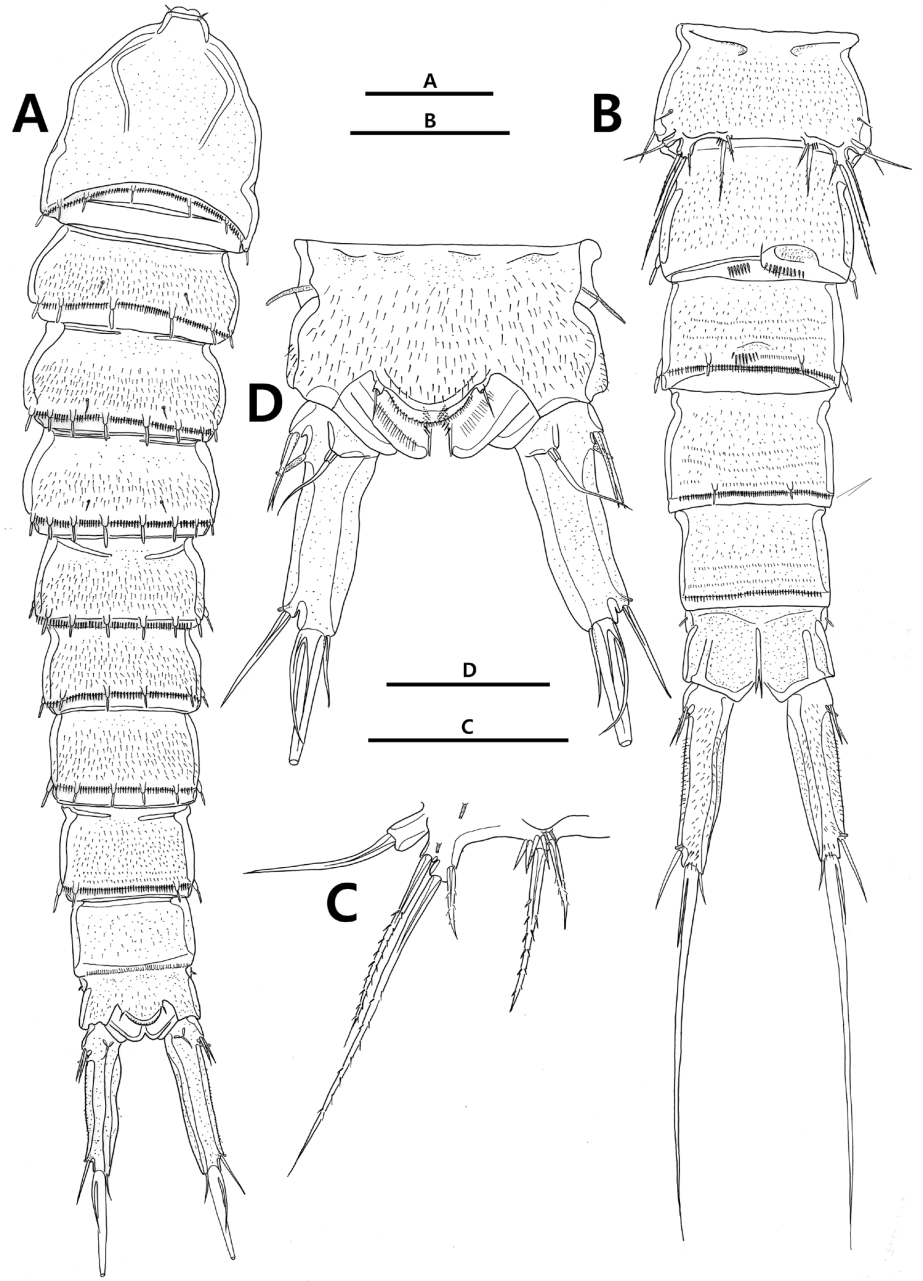
*Strongylacron glabrum* sp. n. **A–C** male: **A** habitus, dorsal **B** urosome, ventral **C** P5. **D** female: **D** anal somite and caudal rami, dorsal. Scale bars 100 µm (**A, B**), 50 µm (**C, D**).

Caudal rami (Figs 6D, 9E, F) cylindrical, tapering posteriorly, as long as anal somite in length, with 2 tube pores and 7 caudal setae: lateral seta I along with seta II inserted in proximal fifth of ramus; seta III half of caudal ramus in length; seta IV small, fused to well-developed seta V at its base; seta V 3.0 times as long as caudal ramus; seta VI shortest, located at inner distal corner; seta VII located in dorsal surface proximally, articulated basally.

Antennule (Figs 3B, 8F) 5-segmented, short, blunt; surface (Fig. 9A) ornamented with small papillae. Segment 1 short, with 3 rows of setules on surface and row of stout spinules on anterior margin. Segment 2 longest. Segments 3 and 5 with aesthetasc fused to seta at its base, respectively. Segment 4 shortest. Setal formula as follows: 1-[1], 2-[8], 3-[7+ae], 4-[1], 5-[10+ae].

**Figure 7.**
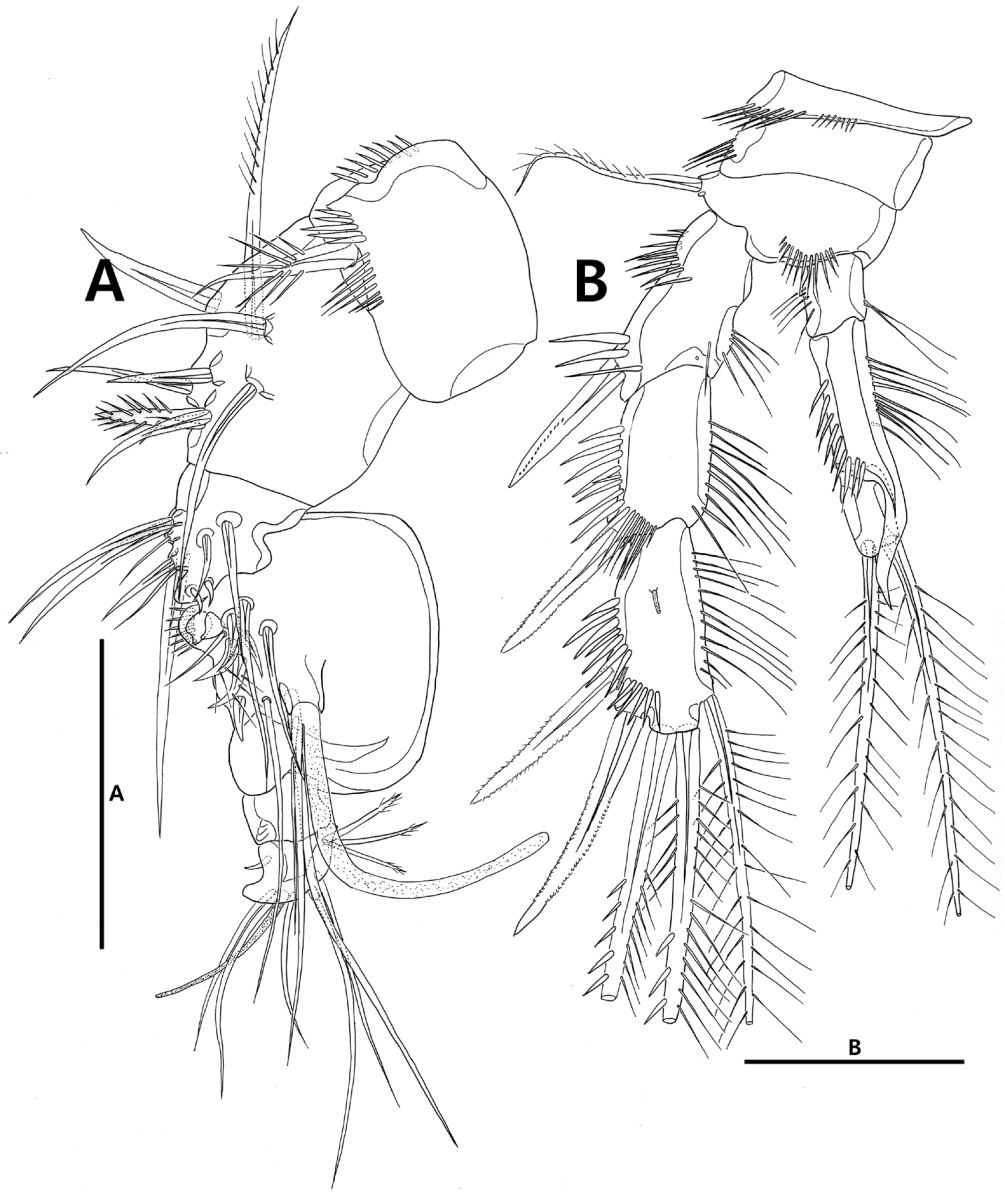
*Strongylacron glabrum* sp. n. male. **A** antennule **B** P3. Scale bars 50 µm.

**Figure 8.**
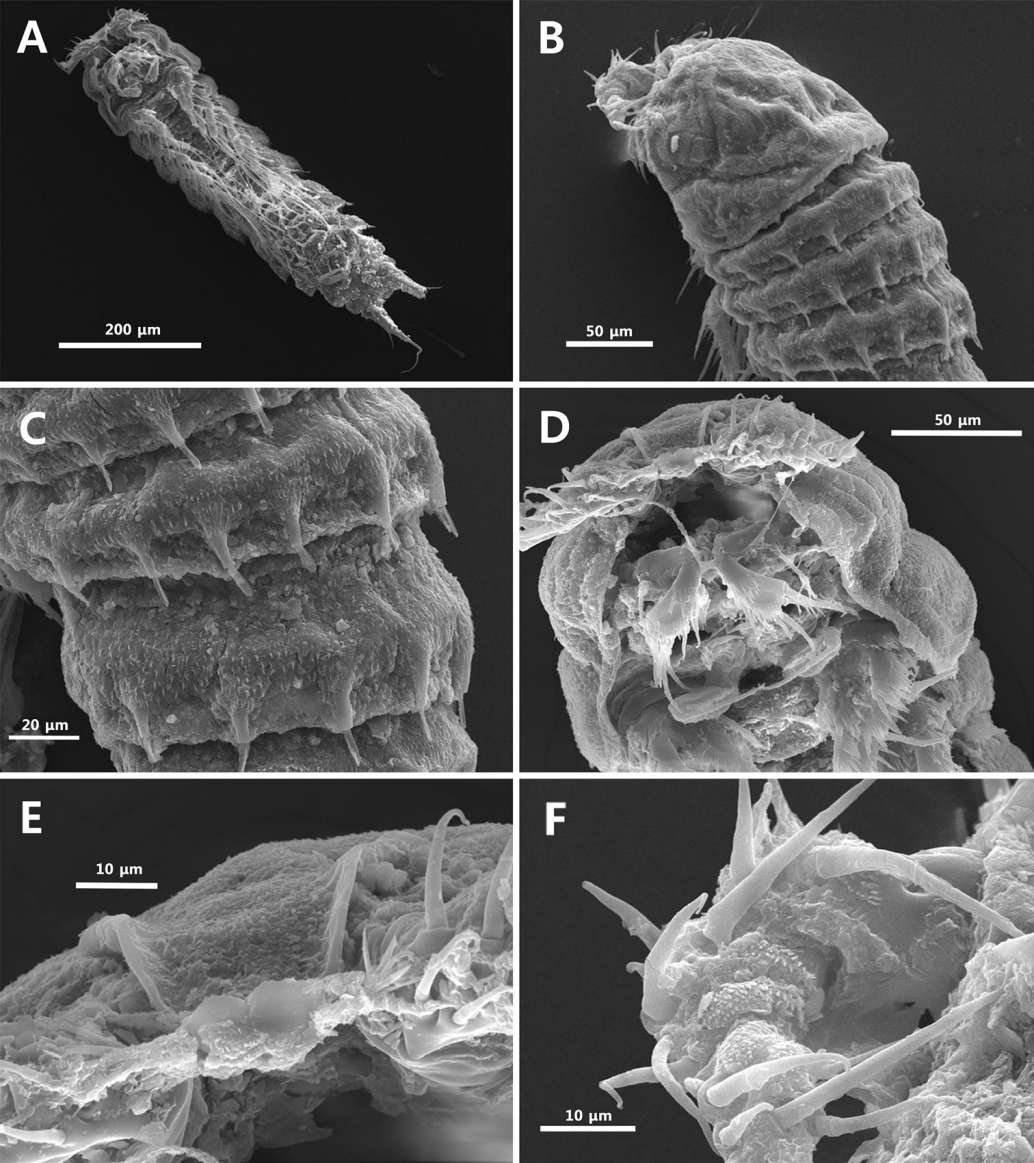
Scanning electron microscope photographs. *Strongylacron glabrum* sp. n. female: **A** habitus, ventral **B** prosome, dorsolateral **C** thoracic somites 2–4, dorsolateral **D** cephalothorax, ventral **E** rostrum, anterior **F** antennules, dorsal.

**Figure 9.**
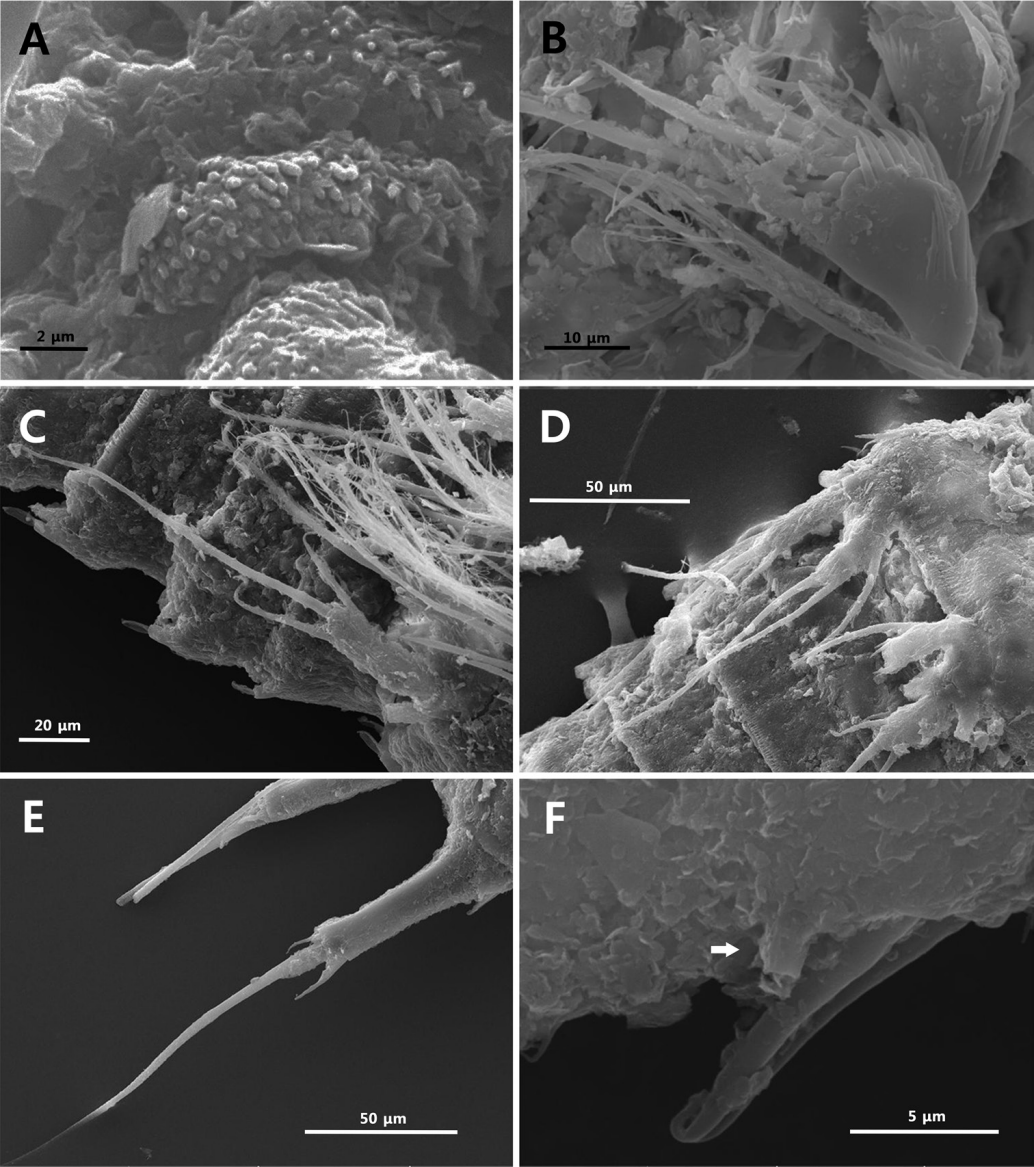
Scanning electron microscope photographs. *Strongylacron glabrum* sp. n. female: **A** surface of antennules, dorsal **B** antennary endopod **C, D** P5 **E** caudal rami **F** caudal setae I, II and tube pore (arrow).

Antenna (Figs 3C, 9B). Coxa small. Allobasis long, with 2 abexopodal setae; antennary exopod small, peduncle-like, with long bipinnate seta. Endopod 1-segmented, slightly shorter than allobasis; anterior margin with row of setules, row of subdistal spinules, and 2 spines; distal margin with 5 non-geniculate spines, 1 slender seta, and 1 tube pore; surface with row of spinules distally.

Mandible (Fig. 3D). Coxal gnathobase well-developed, with 3 bicuspid teeth; outer distal corner broad, rounded; surface with group of setules. Palp 1-segmented; basis with 2 short and 1 long plumose setae; exopod and endopod fused to basis, each represented by plumose seta.

Maxillule (Fig. 3E). Praecoxa with patch of setules on surface; arthrite armed with 5 spines on distal margin, 1 pinnate seta on lateral margin and 2 tube setae on anterior surface. Coxal endite with 1 stout bipinnate and 1 slender setae; surface with row of spinules. Basal endite with 6 elements, 1 row of spinules and 1 row of setules. Both rami incorporated into basis, each represented by 1 plumose and 1 naked setae.

Maxilla (Fig. 3F). Syncoxa with 1 row of setules and 1 patch of setules along outer margin, bearing 2 endites: proximal endite with 2 stout pinnate (one fused to endite proximally) and 1 bare setae; distal endite with 1 pinnate and 2 bare setae. Allobasal endite forming claw like, with 3 elements and 1 tube pore. Endopod incorporated into allobasis and represented by 2 setae. Exopod absent.

Maxilliped (Fig. 3G). Syncoxa with 3 rows of setules and 1 long bipinnate seta. Basis elongate, with 2 rows of setules along palmar margin; outer distal margin with row of setules distally. Endopod represented by claw, longer than length of basis, with accessory seta.

P1 (Fig. 4B). Coxa with row of spinules on anterior surface. Basis with 2 rows of spinules on anterior surface, 1 outer seta, and 1 pinnate inner spine. Exopod 3-segmented, slightly longer than endopod; each segment ornamented with rows of outer spinules and inner setules; exp-1 and -2 with outer spine, respectively; exp-3 with 2 outer spines, 2 apical setae, and 1 posterior tube pore. Endopod 2-segmented; each segment ornamented with rows of outer spinules and inner setules; enp-2 1.5 times as long as preceding one, and armed with 1 short inner seta, 1 long apical seta, and 1 outer spine.

P2–P4 (Figs 4C, 5A, B). Praecoxa with row of spinules on distal margin. Coxa with row of spinules on anterior surface. Basis with 1 or 2 rows of spinules and 1 tube pore on anterior margin, and 1 outer seta. Exopod 3-segmented; each segment ornamented with rows of outer spinules and inner setules; exp-1 and -2 without inner seta; exp-3 with tube pore on anterior margin. Endopod 2-segmented; endopod of P4 very short and slightly exceeding end of P4 exp-1.

Setal formula of P1–P4 as follows:

**Table:** 

	**Exopod**	**Endopod**
P1	I-0, I-0, II,2,0	0-0, I,1,1
P2	I-0, I-0, II,2,0	0-0, 0,2,0
P3	I-0, I-0, II,2,1	0-0, I,2,0
P4	I-0, I-0, II,2,1	0-0, I,1,1

P5 (Figs 5C, 9C, D) distinctly U-shaped, covered with fine setules. Baseoendopod with anterior tube pore and peduncle bearing outer seta; endopodal lobe reaching to 2/3 of exopod, with 3 pinnate setae, 2 tube pores, and 1 row of spinules. Exopod indistinctly separated from baseoendopod, 3.5 times as long as width, with 1 anterior tube pore and 3 pinnate setae; innermost seta shorter than exopod.

##### Male.

**Figure 10.**
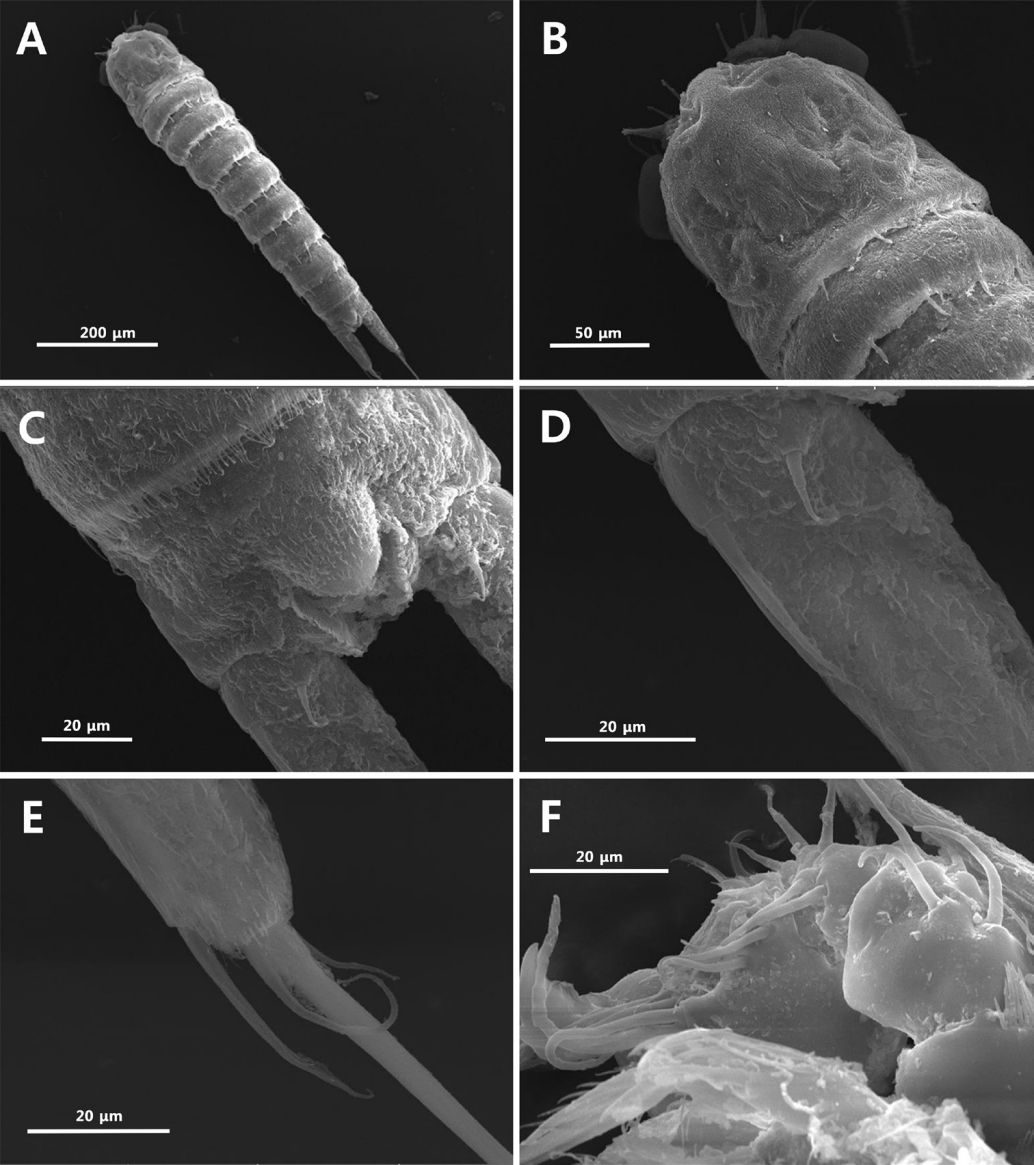
Scanning electron microscope photographs. *Strongylacron glabrum* sp. n. male: **A** habitus, dorsal **B** cephalothorax and thoracic somite 2, dorsal **C** anal somite, dorsal **D** proximal part of caudal ramus **E** distal part of caudal ramus, dorsal **F** antennule, ventral.

Body (Figs 6A, 10A, B) 700.0–934.1 µm (mean 799.8, *n* = 3) in length, measured from anterior margin of rostrum to end of caudal rami.

Urosomites 2 and 3 (Figs 6B, 10A) not fused. P6 (Fig. 6B) asymmetrical, and with 2 rows of setules on posterior margin and plate on one side of body. Urosomite 3, ventral surface asymmetrical ventrally, with 1 row of setules and 1 row of delicate setules.

Caudal rami (Figs 6A, B, 10C–E) longer than female, 1.9 times longer than anal somite in length.

Antennule (Figs 7A, 10F) 6-segmented, subchirocer. Segment 1 with 3 rows of spinules on surface. Segment 4 swollen; proximal corner with 1 row of spinules and 2 spines; small peduncle on inner surface with aesthetasc and seta. Segment 5 shortest with protrusions at distal corner. Each aesthetasc on segments 4 and 6 fused to seta at its base. Setal formula as follows: 1-[1], 2-[8], 3-[10], 4-[12+ae], 5-[0], 6-[9+ae].

P3 (Fig. 7B). Endopod 3-segmented, modified; enp-2 with recurved apophysis at inner distal edge; enp-3 small, with 2 plumose apical setae. Exp-3 with tube pore on anterior surface.

P5 (Fig. 6C). Baseoendopod and exopod confluent. Endopodal lobe very small, with 2 setae, 2 tube pores, and 1 row of spinules; inner seta half of outer one in length. Exopod with 1 tube pore and 3 setae, innermost seta approximately 1/3 of outermost seta in length.

##### Distribution.

The south-western coasts of South Korea.

##### Etymology.

The epithet of the specific name, *glabrum*, is derived from the Latin adjective *glaber*, meaning ‘hairless’ or ‘bare’. This name refers to the naked anterior rostral margin of the new species.

##### Remarks.

Gee and Huys (1996) redefined the taxonomic status of four *Enhydrosoma* species, *Enhydrosoma buchholtzi* (Boeck, 1873), *Enhydrosoma barnishi* Wells, 1967, *Enhydrosoma bifurcarostratus* Shen & Tai, 1965, and *Enhydrosoma vervoorti* Fiers, 1987, belonging to the *buchholtzi*-species group (see Gee 1994), and they established two genera, *Schizacron* Gee & Huys, 1996 and *Strongylacron* Gee & Huys, 1996. These genera share a distinctive U-shaped female P5, which is known as a unique structure of the family Cletodidae T. Scott, 1904, but they are typically divided in terms of the structure of rostrum (Gee and Huys 1996). *Schizacron* is characterized by having a recurved dorsally and markedly bifid anterior rostral margin, while *Strongylacron*’s rostrum is non-recurved and has a broadly rounded anterior margin (Gee and Huys 1996). Additionally, they recognized the presence of a row of fine setules on the anterior rostral margin as a significant generic characteristic of *Strongylacron*. The genus *Strongylacron* was erected based on only one species, *Strongylacron buchholtzi*, with a restricted distribution in the north Atlantic Ocean (northwestern Europe and Canada) (Boeck 1873; Sars 1909; Willey 1929; Wells 1963; Gee and Huys 1996).

In the genus *Strongylacron*, the presence of fine setules on the anterior margin of rostrum seems to be not a generic characteristic but a specific feature to distinguish species due to the discovery of the new species, *Strongylacron glabrum* sp. n., having a naked rostrum. In related genera such as *Cletodes* Brady, 1972, *Enhydrosoma* Boeck, 1873, and *Schizacron* Gee & Huys, 1996, the rostrum of most species are usually naked except for *Cletodes macrura* Fiers, 1991 and *Schizacron barnishi* (Wells, 1967) (Wells 1967; Fiers 1991; Gee 1994; Gee and Huys 1996). There is an additional difference on the position of the tube pore on the caudal rami between *Strongylacron glabrum* sp. n. and the generic diagnosis given by Gee and Huys (1996). The tube pore on outer margin is located proximally in *Strongylacron glabrum* sp. n., while it is inserted medially in the generic diagnosis (Gee and Huys 1996).

Nevertheless, the new species can be placed in the genus *Strongylacron* without doubt based on its possession of the following morphological features: (1) each third exopodal segment of P1–P4 with four, four, five, five setae/spines, (2) rostrum with non-recurved anterior margin dorsally, (3) a minute antennary exopod with a pinnate seta, (4) female P5 distinctly U-shaped, (5) a recurved apophysis on the second endopodal segment of male P3, and (6) two apical setae on the third endopodal segments of male P3.

*Strongylacron glabrum* sp. n. shows many differences from *Strongylacron buchholtzi*, including: (1) the anterior margin of the rostrum is slightly concave in the middle and naked between sensilla (vs. having row of fine setules (Sars 1909, pl. CXCVIII, R + a1.; Gee and Huys 1996, Fig. 1B)); (2) the outermost seta on the distal margin of the antennary endopod is stout as neighboring one (vs. slender than the neighboring one (Sars 1909, pl. CXCVIII, a2.; Gee and Huys 1996, Fig. 1C)); (3) the mandibular basis has one long and two short setae (vs. two long and one short setae (Gee and Huys 1996, Fig. 3B)); (4) the mandibular gnathobase does not have one plumose seta (vs. having a short and stout pinnate seta (Gee and Huys 1996, Fig. 3B)); (5) caudal seta VI is shorter than seta IV in length (vs. seta VI is longer than seta IV (Sars 1909, pl. CXCVIII, F.; Gee and Huys 1996, Fig. 2A)); (6) the tube pore on outer margin of caudal rami is proximally inserted (vs. medially (Gee and Huys 1996, Fig. 2A)); (7) the length to greatest width ratio of caudal ramus in male is about 3.6:1 (vs. 2.6:1 (Gee and Huys 1996, Fig. 2B)); (8) the length to greatest width ratios of female P5 exopod and endopodal lobe are approximately 3.5:1 and 2.7:1, respectively (vs. at most 3.0:1 and 1.8:1, respectively (Sars 1909, pl. CXCVIII, p5.; Gee and Huys 1996, Fig. 3D)); (9) the innermost seta on female P5 exopod is shorter than the length of exopod (vs. longer than exopod (Sars 1909, pl. CXCVIII, p5.; Gee and Huys 1996, Fig. 3D)); (10) the outermost seta on the endopodal lobe of male P5 is reaching half of the middle one on exopod (vs. slightly shorter (Gee and Huys 1996, Fig. 4C)); (11) each posterior border of the prosomite has eight or ten rod-like projections (vs. 14–18 (Gee and Huys 1996, Fig. 1C)) (Table 1).

**Table 1.** Morphological differences between *Strongylacron buchholtzi* and *Strongylacron glabrum* sp. n.

Character	*Strongylacron buchholtzi*	*Strongylacron glabrum* sp. n.
Body		
length (µm)♀	500–810	889–923
Prosomite		
number of rod-like projections on posterior border	14–18	8–10
Rostrum		
anterior margin	with row of fine setules	naked
Antenna		
outermost seta on distal margin of endopod	slender	stout
Mandible		
gnathobase	with seta	without seta
basis	with 2 long, 1 short	with 1 long, 2 short
P5	
exopod, length ratio to greatest width♀	at most 3.0:1	approximately 3.5:1
endopodal lobe, length ratio to greatest width♀	at most 1.8:1	approximately 2.7:1
length of outer seta to baseoendopod to middle seta on exopod ♂	slightly short	half
Caudal ramus		
position of tube pore on lateral margin♀	median	proximal
seta III, length ratio to caudal ramus♀	1.1:1	0.4:1
seta VI, length ratio to caudal ramus♀	0.84:1	0.3:1
length ratio to greatest width ♂	2.6:1	3.6:1

Members of the family Cletodidae are known as mud-burrowers from shallow and sublittoral marine habitats (Por 1986; Boxshall and Halsey 2004; Kim et al. 2014; Song et al. 2014). *Strongylacron* species were also reported from muddy bottoms. *Strongylacron buchholtzi* was known from intertidal and sublittoral (depth of 20 m) habitats on the Atlantic Ocean (northwestern Europe, Canada) (Boeck 1873; Sars 1909; Willey 1929; Wells 1963; Gee and Huys 1996). *Strongylacron glabrum* sp. n. was found from intertidal mudflats on the south-western coasts of South Korea. The genus *Strongylacron* herein is first recorded from the Pacific Ocean.

### Key to known species of the genus *Strongylacron*

**Table:** 

1	Rostrum furnished with densely fine setules on anterior margin; female P5 exopod and endopodal lobe at most 3.0 and 1.8 times as long as greatest width, respectively; each prosomite in female with 14–18 rod-like projections	***Strongylacron buchholtzi* (Boeck, 1873)**
–	Rostrum naked between sensilla on anterior margin; female P5 exopod and endopodal lobe approximately 3.5 and 2.7 times as long as greatest width, respectively; each prosomite in female with 8–10 rod-like projections	***Strongylacron glabrum* sp. n.**

## Supplementary Material

XML Treatment for Strongylacron glabrum
